# Spatio-temporal process and influencing factors of polluting enterprises' migration: an empirical study based on the Yangtze River Delta

**DOI:** 10.1038/s41598-023-40621-8

**Published:** 2023-08-17

**Authors:** Bingquan Lin

**Affiliations:** 1 Department of Economics and Management, Jiangsu College of Administration, Nanjing, 210009 China; 2 School of Urban and Regional Sciences, East China Normal University, Shanghai, 200241 China

**Keywords:** Environmental impact, Environmental economics

## Abstract

The migration of polluting enterprises (MPE) is a crucial subject within the fields of Environmental Economic Geography and Enterprise Geography. An analysis was conducted on the temporal-spatial evolution and influencing factors of the MPE by employing the full-sample enterprises data from the "Qichacha" system. The main findings were obtained: (1) The polluting enterprises in the Yangtze River Delta have undergone an evolutionary process of proximity migration, intra-provincial proximity to intra-provincial long distance migration, with intra-provincial migration being the main focus supplemented by inter-provincial migration, and coexisting intra-provincial and inter-provincial migration. (2) The estimation results, based on the panel negative-binomial model, reveal that the variables having a greater impact on the MPE encompass environmental regulation, industrial upgrading policies, cost factors, geographical distance, coastal and inter-provincial boundaries, etc. These influencing factors display time heterogeneity and industry heterogeneity. (3) By taking the Nijiaxiang Group, which was involved in the 321 Explosion Accident in Xiangshui, as an example, it was discovered that insufficient inheritance of environmental protection concepts, corruption of officials, and social governance problems have resulted in shortcomings in the area. This has led to a lowered level of local environmental regulation, transforming the local area into a haven for polluting enterprises such as the Nijiaxiang Group. The contribution of this study lies in observing the MPE from the perspective of "inter-regional association," and within the group enterprises. It addresses the deficiencies of existing research that mainly examines the evolution of industrial geographic patterns and statically observes polluting enterprises' entry and exit behavior. Moreover, this study demonstrates that the Pollution Shelter Hypothesis is not universal and has an impact in the Yangtze River Delta region after 2012.

## Introduction

The migration of industries that contribute to pollution has emerged as an issue of critical importance and immediacy. Since the convening of the 18th National Congress of the CPC, the country has taken on an active role in meeting its obligations as a major nation by adhering to its commitments with respect to reducing emissions. The CPC has set forth the overarching objective of promoting "synergies in reducing pollution and carbon emissions", ultimately achieving victory in the war against pollution prevention and control. However, at the local level, the migration of polluting industries has emerged as a key strategy for reducing pollution and carbon emissions in certain areas. This has resulted in the widespread migration and migration of polluting enterprises, particularly with regard to equity investment-based spatial migration, which has largely escaped the attention of academia, local authorities, and the general public.

In this context, regions with comparatively lax environmental regulations may become "pollution havens," subjecting their inhabitants to severe risks of pollution exposure and health problems. This has led to the emergence of "cancer villages" in some areas. In the early twenty-first century, a county located in northern Jiangsu famously declared that "it is better to die from poisoning than to die from poverty!" More recently, certain industrial parks in Anhui have expressed their eagerness to "contact" local high-pollution and high-emission enterprises that were eliminated from a chemical industry park situated in Jiangsu. Given the differential development strategies adopted across various regions, the pollution industry has undergone spatial reconfiguration, and pollution enterprises have also pursued distinct pathways for migration.

The migration of polluting enterprises represents a form of migration that has been extensively researched in the field of environmental economic geography and enterprise geography. The extant literature has mainly studied this phenomenon from three perspectives. (1) Scholars have explored the changes in the geographical pattern of pollution industries by analyzing the shifts in the scale and structure of polluting enterprises^1,2^. This approach, however, has been limited by the absence of actual industrial flow data between regions, leading to the issue of "decreasing in one place, but not necessarily increasing in the other". This has impeded efforts to accurately identify the actual movement patterns of polluting industries between regions. (2) Some scholars have conducted field research and case analysis to examine specific regions, enterprises, or listed enterprises^3^, yet it remains challenging to analyze the migration trajectories of various polluting enterprises as a whole. (3) Due to the absence of relational data, previous research has divided the migration process of polluting enterprises into their entry and exit behaviors, with a focus on the impact of "regionalism" factors on these behaviors^4^. However, it is difficult to investigate the flow patterns of enterprises between regions, thereby limiting the exploration of the influence of inter-regional association factors.

For MPE, the Yangtze River Delta region has a strong representation. The Yangtze River Delta is one of the most densely populated manufacturing regions in contemporary China. Since 2001, the Yangtze River Delta region carried out the preliminary exploration of the policy, and industrial migration became an important measure to promote regional coordinated development. There has been a gradual rise in inter-regional investments made by enterprises. As a result, the migration of polluting industries has become more prevalent. To address this phenomenon, this study focuses on the Yangtze River Delta as a research subject, using the complete sample of enterprise migration data provided by the "Qichacha" database. Employing both quantitative modeling and qualitative analysis, this research aims to explore the spatio-temporal processes and underlying mechanisms of polluting enterprise spatial migration. This study not only meets the practical demand of analyzing the migration path and dynamic mechanisms of polluting enterprises in the new era, but also fulfills the theoretical requirement of promoting the integration of environmental economic geography, enterprise geography, relational economic geography, and other relevant sub-disciplines.

## Literature review and a theoretical framework

### The migration of polluting enterprises under the organization mode of "multi-sector enterprises"

The primary methods for polluting enterprises' migration encompass overall migration, Establish subordinate enterprises, and mergers and acquisitions^3,5,6^ (Table 1). Certain scholars have also suggested that the migration of production bases and process outsourcing constitute additional migration behaviors of polluting enterprises^7,8^. However, the production base's migration is still effectuated through equity investments and mergers and acquisitions, and there is no inevitable subordination relationship between the process outsourcing partner and the enterprise entity; rather, a business cooperation relationship exists, which should not be classified as the migration behavior of polluting enterprises. Presently, group enterprises characterized by "multi-sector, multi-location, and multi-value chain" have emerged as the predominant spatial organization form for enterprises^9^. Group enterprises can attain migration by extend specific industrial links in alternate locations by establishing branches or holding subsidiaries. Establishing subordinate enterprises has become the most important way to MPE. Therefore, this paper analyzes the MPE by using the data of enterprises establishing subordinate enterprises.

**Table 1:** Main types of the MPE.

Migration method	Migration content
Overall migration	Two scenarios occur: ① The name of the enterprise remains unchanged, but the registration location is changed; ② Investors close the original enterprise and register a new enterprise in other areas, resulting in changes to both the name and registration location of the enterprise
Establish subordinate enterprises	Enterprises extend specific industrial links in alternate locations by establishing branches or holding subsidiaries
Merger and acquisition	Enterprises employ capital to procure equity in other enterprises through acquisitions, mergers, etc., thereby establishing subsidiaries in various locations through participation or ownership

Data source: collated according to relevant research^3,5,6^.

At present, this phenomenon of "pollution migration within group enterprises" has emerged at the forefront of academic focus. Rijal and Khanna discovered that pollution substitution behavior exists within group enterprises in the United States, indicating that if a compliant factory possesses a "sister factory" in the same industry, the latter's toxic gas emissions will escalate by approximately 35–56%^10^. Domestic scholars in the field of environmental economics have promptly responded, positing the existence of "pollution havens" within group-type enterprises, with enterprises situated in areas with elevated pollution charges transferring a portion of their production to enterprises in regions with lower pollution charges^11^. Examining investment quantity and geographical distance, the enhancement of local environmental regulation of the parent enterprise results in a significant increase in the number of subsidiaries in disparate locations, with a more dispersed distribution and an extended distance between the parent enterprise and its subsidiaries^12^. However, due to the absence of "relational data" at the enterprise level, such as investment, equity, and branch companies, the majority of studies are unable to scrutinize the group background underpinning the enterprise, causing inter-enterprise investment connections and value chain divisions to become the "black box" of research within this field. Domestic scholars predominantly depend on data from publicly-traded enterprises to advance investigations in this domain. However, such enterprises are stringently regulated in terms of information disclosure, environmental monitoring, and other aspects, thereby possessing relatively superior pollution control capabilities^11,13^. Conversely, the academic sphere necessitates the execution of extensive sample analyses, particularly those involving enterprises exhibiting the attributes of being "small, scattered, chaotic, and dirty."

### Local government factors and the migration of polluting enterprises

Numerous factors have been proposed in previous studies to explain the migration of polluting enterprises from various perspectives, such as government and policy, industrial development environment, and geographical location. Notably, government and official factors encompass a range of considerations, including environmental regulation, industrial upgrading, official promotion, and counterpart assistance, etc.

In the academic community, it is widely accepted that environmental regulation policies are the primary driving force behind the migration of polluting enterprises. As regions engage in a "race to the bottom," variations in the stringency of environmental regulations can be observed^12^. Consequently, polluting enterprises tend to gravitate towards regions with less rigorous environmental standards. Nevertheless, the role of environmental regulation is multifaceted. Some studies suggest that the influence of environmental regulation on the MPE exhibits a U-shaped pattern^14^ or an inverted N-shaped pattern^15^. In addition, there is a threshold effect of environmental regulation: when regional economic development is low, stricter environmental regulation can stifle the inflow of polluting enterprises. However, once environmental regulation reaches a specific threshold, technological innovation within enterprises will be spurred, attracting more enterprises to relocate^16^. Secondly, industrial upgrading is a crucial factor in the MPE. As the Yangtze River Delta region moves towards late industrialization and post-industrialization, cities are experiencing a new phase of economic transformation and industrial upgrading. Local governments have placed significant emphasis on industrial upgrading policies, but the nature of these policies varies depending on the stage of regional development. Central cities such as Shanghai, Suzhou, Hangzhou, and Nanjing exhibit more robust industrial upgrading, while less developed areas tend to have weaker industrial upgrading efforts. Consequently, regions that prioritize industrial upgrading are better equipped to transfer polluting industries to less developed areas. Thirdly, in the context of fiscal decentralization, the current performance appraisal system incentivizes local officials to prioritize local economic development as a means of achieving political success. This approach may "favor" polluting enterprises and impede efforts to relocate them^17,18^, or even attract high-polluting industries to increase tax revenue. Nevertheless, as environmental considerations are gradually integrated into the assessment metrics of local governments, the promotion system may exert a nuanced impact on the movement of polluting enterprises. This paper introduces the characteristics of officials in both the origin and destination regions to measure their influence on the migration of polluting enterprises. Finally, government-to-government assistance policies can facilitate the rapid integration of enterprises into local networks and lower operating costs. To explore the impact of this policy, this paper includes a virtual variable of matching assistance between cities in the Yangtze River Delta region.

### Industrial development environment, geographical location and the migration of polluting enterprises

1. The spatial migration of polluting enterprises will consider both costs and benefits^19^. According to existing studies, the flow direction of such enterprises is influenced by a variety of factors, including but not limited to industrial agglomeration, labor costs, export environment, urban innovation capacity, and level of economic development^20^. In addition, the phenomenon of "lock-in effect" stemming from industrial agglomeration serves to impede the migration of these polluting enterprises^7,21^.
2. Geographical location factors include regionalism and inter-regional association factors. Regionalism comprise a variety of elements, such as river line, port advantage, resource endowment, and regional boundary^22^. Polluting enterprises within a city tend to aggregate from the central urban area towards the suburban and border regions^23^. In a province, such enterprises tend to flow towards border cities and show a particular preference for the downstream areas of significant river systems^24^. Moreover, the multidimensional proximity between inflow and outflow areas is also a salient factor that impacts the migration of polluting enterprises^25,26^. The migration of polluting enterprises is facilitated by shorter geographical, cultural, and institutional distances between locations. Similar institutional arrangements can significantly reduce transaction costs for these enterprises. Despite this, there has been a notable lack of research focused on inter-regional associations in existing studies.

**Figure 1:**
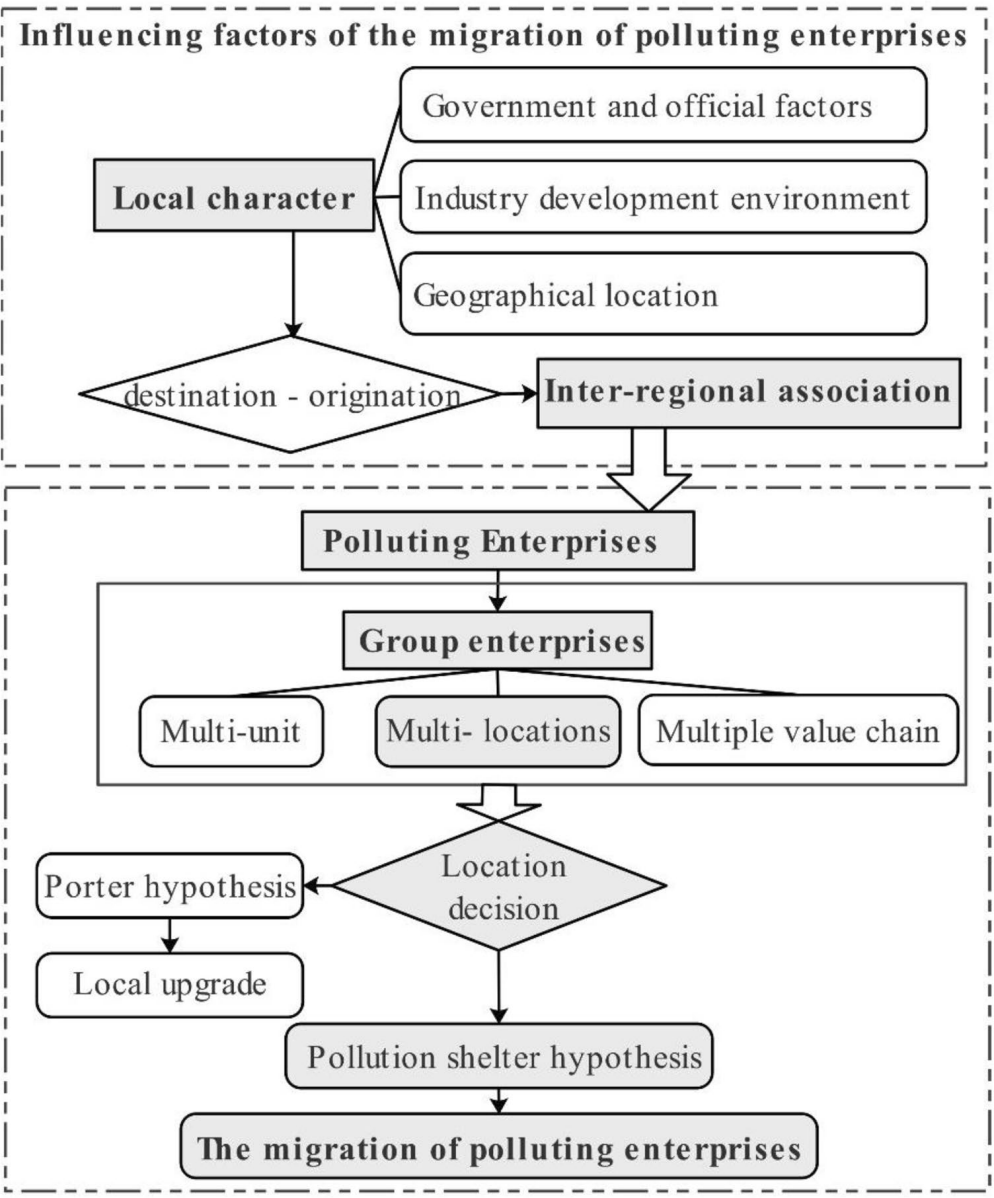
Research framework.

This paper argues that it is necessary to analyze the MPE from a multi-scale perspective (Fig. 1): (1) From the perspective of interregion, it implies that the MPE is a network of "flow space". This network is composed of the investment and industrial flows from the source to the destination. The mobility of this network is influenced by the regionalism of both the source and the destination, as well as the interregional correlation. (2) From the perspective of individual enterprises, the migration of enterprises occurs within the spatial organization of "group enterprises". Enterprises within this kind of spatial organization, characterized by multi-sectors, multi-locations, and multi-value chains, have the ability to make location selections by comprehensively considering the location factors. (3) From the perspective of the source of polluting enterprises, it reveals that the location decisions of such enterprises are diversified under the combined influence of both internal and external factors. These factors include: firstly, upgrading and transformation within their local regions; secondly, migration to other cities; and thirdly, the realization of migration through the establishment of holding subsidiaries and branches.

In conclusion, the MPE is significantly influenced by both regionalism and inter-regional association. While existing studies have predominantly focused on environmental regulation, they have not provided adequate analysis on regional comprehensive factors^27^, nor have they thoroughly analyzed inter-regional relevance factors. This paper aims to address this gap by exploring the spatio-temporal evolution and influencing factors of the MPE from the perspective of "inter-regional association".

## Data processing and analysis methods

### Data source

1. The source of enterprise directory data and inter-enterprise investment relationship data primarily originates from the information gathered between 2001 and 2018 within the "Qichacha" enterprise credit information inquiry platform (www.qcc.com, henceforth referred to as "Qichacha Database"). The database furnishes details such as the enterprise's denomination, the legal representative's moniker, the consolidated social credit code, the establishment's locale, the inception date, the operational status, the industry classification, the principal commerce, the registered funds, the equity percentage, the shareholder particulars, the counterparty investment enterprise, the investment sum, the ultimate beneficiary, and the intellectual property rights, etc.
2. The statistical data pertaining to economic and social indicators at the city level, such as industrial wastewater and SO2 emissions, gross industrial output value, total exports, per capita wage, average land transfer price, and others, were predominantly obtained from the statistical yearbooks of provinces and cities, China Land Resources Yearbook, EPS Chinese city database, among other credible sources. Moreover, local dialect data were sourced from the Attribution of Dialects in China's Counties^28^. The attribute data of local officials, on the other hand, were extracted from the China Political Elite Database (CPED)^29^. Finally, the author collected the data on pairing assistance between cities by referring to the official documents of each region.

### Defining polluting enterprises

The polluting enterprise is broadly defined as an enterprise that generates a considerable amount of pollutants directly or indirectly during production processes. Such pollution poses a threat to human beings, animals, plants, and overall environmental degradation if not managed and controlled appropriately^30^. To identify polluting enterprises, a commonly adopted approach involves first defining the polluting industry and subsequently designating all enterprises belonging to said industry as polluting enterprises.

At present, the methods for defining industries that are responsible for polluting the environment include various approaches: (1) The legal approach, which relies on official documents issued by the state and relevant ministries to identify polluting industries. Examples of such documents include the Notice on Environmental Protection Verification for Listed Enterprises Applying for Listing and Listed Enterprises Applying for Refinancings issued by the Ministry of Environmental Protection, the Environmental Information Disclosure Guide for Listed Enterprises, and the First National Pollution Source Census Plan issued by The State Council^31,32^; (2) the total amount or intensity method sets a pollution emission threshold to select polluting industries based on either their total amount or intensity of pollution^33,34^; (3) the cost method selects polluting industries based on the proportion of pollution control costs in the total cost^35^. This study has identified 23 industrial categories as polluting industries based on the aforementioned methods. These industries include: coal mining and washing industry, petroleum and natural gas mining industry, ferrous metal ore mining industry, non-ferrous metal ore mining industry, non-metallic ore mining industry, mining professional and auxiliary activities, other mining industry, agricultural and sideline food processing industry, food manufacturing industry, wine, beverage and refined tea manufacturing industry, textile industry, leather, fur, feathers and their products and footwear industry, paper and paper products industry, petroleum, coal and other fuel processing industry, manufacturing of chemical raw materials and chemical products, pharmaceutical manufacturing, chemical fiber manufacturing, rubber and plastic products industry, non-metallic mineral products industry, ferrous metal smelting and rolling processing industry, non-ferrous metal smelting and rolling processing industry, metal products industry, and electric power and thermal production industry.

### Identify the MPE

In this paper, the establishment of cross-regional subsidiary companies based on green field investment, mergers and acquisitions is regarded as the enterprise migration behavior. The steps for the identification of this behavior are specified as follows:

1. A list of enterprises operating within 23 polluting industries located in the Yangtze River Delta region was compiled from the Qichacha database.
2. Information regarding foreign holding investments, with a share ratio greater than 50%, as well as subsidiary information for each of the aforementioned enterprises was collected. It is noteworthy that each polluting enterprise may establish multiple holding subsidiaries and branches across different regions.
3. This study transformed the aforementioned "one-to-many" investment relationships into a "one-to-one" migration relationship for polluting enterprises. As a result, a total of 5,840 migration sample were identified in this study.
4. The paper summarized the "relationship pairs" of the aforementioned enterprises and transformed them into "relationship pairs" of cities with districts. This allowed for the creation of a directed correlation network comprised of 41 cities with districts as nodes, where the frequency of polluting enterprise migration between cities with districts was used as the weight for the network.

### Model setting and variable selection

This study focuses on the impact of inter-regional association factors on the MPE. In the region of the Yangtze River Delta, a relationship matrix among the districted cities was established. The explained variable was the frequency of the MPE between cities, while explanatory variables included urban attributes and various relational variables between cities. It is necessary to adopt a counting model because the explained variables are integers. Poisson model and negative binomial model are both common counting models. One of the original assumptions of the Poisson model is that the expectation of the explained variable is equal to the variance. However, after conducting calculations, it was found that the variance of the sample utilized in this study is approximately three times that of the mean value (mean value is 0.185, variance is 0.685), indicating that the explained variables exhibit the phenomenon of "excessive dispersion". Results from the negative binomial regression model reveal that the confidence interval of alpha is (1.654, 2.061), and at the significance level of 1%, it is possible to reject the null hypothesis of an overly dispersed parameter "alpha = 0". As a result, this study adopts the negative binomial regression model.

A matrix of the assistance pairs among cities in the Yangtze River Delta region was formulated, and the assistance pairs as the study samples. Regression analysis was conducted through a negative binomial model, in which the frequency of the MPE between cities were dependent variable , and various variables of the relationship between cities were used as the explanatory variables. The baseline model is as follows:

1 $${MPE}_{i,t}={\beta }_{0}+{\beta }_{1}{X}_{i,t-1}+{\mu }_{t-1}+{\varepsilon }_{c,t-1},$$

where *i* indicates an assistance pair, *t* is the year, *MPE* is the dependent variable, *X* represents a series of explanatory variables, *μ*_*t*_ is the time attribute, *ε*_*i,t*_ is a random error term, *β*_0_ is a constant term, and *β*_1_ is coefficients of *X*.

Premised on the aforementioned theoretical analysis, this paper primarily examines the impact of policy and official factors, industrial development environment, and geographical location on the MPE. Table 2 outlines the measurement methods, value methods, and key functions of explanatory variables.

**Table 2:** Independent variables and explanations.

Type	Variable	Measurement method	Value method
Policy and official factors	Environmental regulation intensity	The reciprocal of comprehensive emission coefficient is shown in formula (1), (2)	I
	Industrial upgrading policy	The proportion of lightly polluting industrial enterprises;	I
	The characteristics of strong incentive officials from origin	Whether the secretary of the CPC Municipal Committee is under the age of 55 and is not a member of the Standing Committee of the Party Committee of a higher administrative unit. If the value is 1, otherwise it is 0	II
	The highly motivated official characteristics of the destination		II
	Pair assistance policies	Whether there is a reciprocal helping relationship between the two cities? If yes, the value is 1; otherwise, it is 0;	II
Industry development environment	Total output value of industry above designated size	Original value	I
	Average unit price of land sale	Original value	I
	Labor cost	Average wages of employees at the city level;	I
	Regional export intensity	original value	I
	Concentration level	The number of polluting enterprises adopted within the city;	I
	Urban development intensity	The proportion of construction land in the total area of the administrative area;	I
	Regional innovation ability	Number of all invention patents granted;	I
Geographical location	Geoproximity	Geographical distance	II
	Institutional proximity	Whether in the same province	II
	Cultural similarity	If the two cities belong to the same dialect area, the Cultural similarity is 1; otherwise, it is 0	II
	Terrain difference	Average slope at city level;	I
	Port advantage	If the city has a port, the value is 1; otherwise, it is 0	I
	Inter-provincial boundary city	If the city belongs to Inter-provincial boundary city or coastal city, the value is 1; otherwise, it is 0;	I

Value method: I is the value of the destination minus the value of the source, and II is the original value of the variable.

Factoring in relevant studies^36,37^, the level of environmental regulation is measured by the reciprocal of the comprehensive emission coefficient of pollutants. The specific calculation steps are as follows:

1. In this study, linear standardization of industrial wastewater and industrial sulfur dioxide emissions per unit of output value for each city was conducted. This paper mainly calculated as follows: [However, while industrial dust emissions were included in some studies, due to the serious issues of missing data on industrial dust, this paper did not incorporate the emissions to ensure the integrity of the sample].
2. 2 $${PE}_{ij}^{*}=[{PE}_{ij}-\mathrm{min}({PE}_{j}) ]/[\mathrm{max}({PE}_{j})-\mathrm{min}\left({PE}_{j}\right)],$$
  where, *PE*_*ij*_ is the pollutant discharge per unit output value of *j* pollutants in city *i,* max(*PE*_*j*_) and min(*PE*_*j*_) are the maximum and minimum values of each indicator in all cities, and ${PE}_{ij}^{*}$ is the standardized value of the indicator.
  To account for the variations in pollutant characteristics arising from the dissimilarities in emission types across different cities, an adjustment coefficient was employed. Specifically, the adjustment coefficient is ${W}_{j}={PE}_{ij}/{\overline{PE} }_{ij}$, where ${\overline{PE} }_{j}$ is the urban average level of *j* pollutant emissions per unit output value during the sample period.
3. Calculate the combined emission coefficient at the city level:
  3 $${El}_{i}= \frac{1}{2}\cdot {\sum }_{i=1}^{2}{W}_{j}{\cdot PE}_{ij}^{*}.$$
  ${EI}_{i}$ is the comprehensive emission coefficient of pollutants, representing the comprehensive pollutant intensity to be discharged per unit of output value. This variable captures the extent to which pollution emissions are tolerated during the production process, with higher values of ${EI}_{i}$ indicating more lenient local environmental regulations. To measure the strength of environmental regulations, the reciprocal of ${EI}_{i}$ was utilized. In addition, the difference in environmental regulation intensity between the source and destination regions was measured by computing the difference in their respective regulation intensities.

## Spatio-temporal evolution characteristics of the MPE

The provinces such as Jiangsu, Zhejiang, and Shanghai have witnessed a substantial increase in investments in "village enterprises", "township enterprises", and "hot development zones" after 1978. Since 2001, the policy of regional coordinated development has become an important part of China's regional development strategy. The Yangtze River Delta region carried out the preliminary exploration of the policy, and industrial migration became an important measure to promote regional coordinated development. There has been a gradual rise in inter-regional investments made by enterprises. As a result, the migration of polluting industries has become more prevalent. In addition, the Covid-19 pandemic broke out at the end of 2019, and the impact mechanism behind the MPE is more complex after this time. Therefore, this study analyzes the MPE from 2001 to 2018.

**Figure 2:**
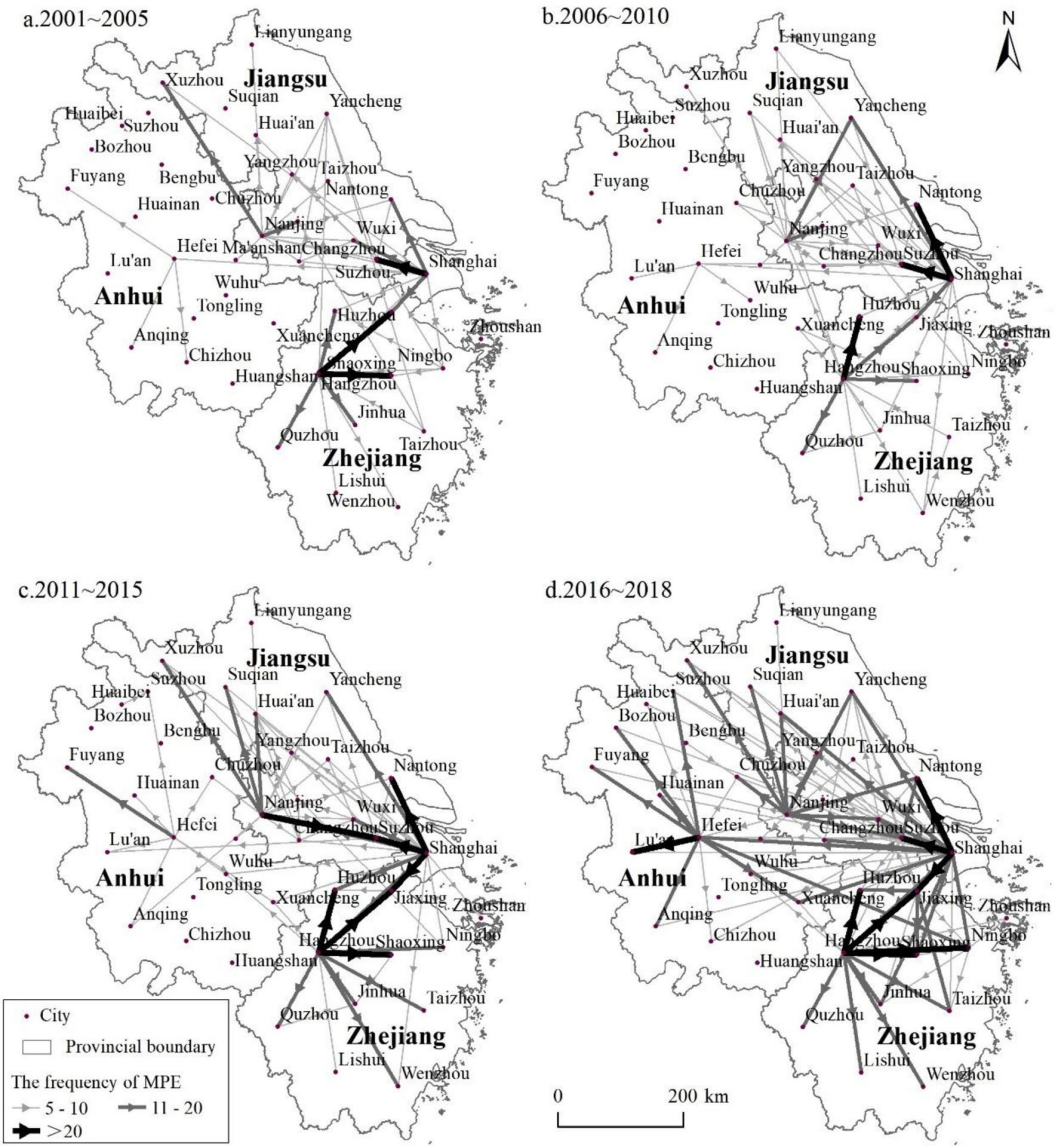
Flow chart of the MPE from 2000 to 2018. This image was created by the author using ArcGIS 10.6.

This study provides a summary and analysis of the historical context and spatial–temporal evolution of polluting enterprises' migration in the Yangtze River Delta region after the year 2000. To facilitate a comprehensive analysis, the region comprising Jiangsu, Zhejiang, and Anhui provinces has been further divided into sub-regions, namely Southern Jiangsu, Central Jiangsu, Northern Jiangsu, Eastern Zhejiang, Northern Zhejiang, Southwest Zhejiang, Southern Anhui, Central Anhui, and Northern Anhui (Fig. 2).

From 2001 to 2018, the migration trajectory of polluting enterprises in the Yangtze River Delta underwent an evolutionary process characterized by the evolution process of: "adjacent migration—intra-provincial adjacent migration to intra-provincial long-distance migration—emphasizing intra-provincial migration, supplemented by inter-provincial migration—parallel intra-provincial and inter-provincial migration."

1. From the perspective of migration direction, it has been observed that the migration of polluting industries has been taking place in the regions of Jiangsu and Zhejiang for an extended period. Moreover, the polluting industries in Shanghai have been continuously expanding into Jiangsu. It was after 2000 that the pollution enterprises in Shanghai started strengthening their migration into Zhejiang, while the pollution enterprises in Zhejiang also intensified their migration into Jiangsu. Jiangsu is an area with relatively lower costs of land and power supply compared to Zhejiang, which has made it a favorable destination for enterprises from both Shanghai and Zhejiang to relocate. Furthermore, the migration of polluting industries from Zhejiang and Jiangsu into Anhui has continued to increase after 2005. Specifically, for the polluting enterprises in Zhejiang, their migration frequency in the southern region of Anhui is higher than that in both southern and northern Jiangsu.
2. Regarding the source and destination of enterprise migration, Shanghai serves as the primary source, while Nanjing, Suzhou, Hangzhou, Hefei, and other provincial capitals or central cities have successively emerged as significant sources of polluting industries. Analyzing by province (city), Shanghai's primary migration occurred in southern Jiangsu and northern Zhejiang before 2005. However, in the later period, there was a rapid increase in the number of polluters expanding towards northern Jiangsu, central Jiangsu, eastern Zhejiang, and northern Anhui. After 2005, the primary migration flow of the Yangtze River Delta shifted from southern Jiangsu to northern Jiangsu. Additionally, the migration of southern Jiangsu towards southern and northern Anhui witnessed a gradual increase after 2010. Anhui province has experienced intra-provincial migration for a prolonged duration, with the primary flow being from central Anhui to northern Anhui. As for Zhejiang enterprises, they mainly operate in northern Zhejiang and expand towards Jiaxing, Huzhou, and other areas from Hangzhou. After 2010, they expanded their operations to Wenzhou, Ningbo, Lishui, and other areas in eastern and southwestern Zhejiang.
3. Regarding policy background, the migration trajectory of polluting enterprises exhibits a strong correlation with policies aimed at industrial upgrading, environmental regulation, and matching assistance. ① Industrial upgrading policy. Jiangsu has put forward "Jiangsu Traditional Industry Upgrading Plan", "South Jiangsu National Independent Innovation Demonstration Zone" and other policies to encourage enterprises in South Jiangsu to accelerate the "north–south industrial gradient transfer" by relocating and transforming their operations. Similarly, Zhejiang has implemented policies such as the "Return of Zhejiang Businessmen" and the "Huzhou Provincial Industrial Transfer Demonstration Zone." Moreover, Shanghai has announced its "Key Work Arrangement of Industrial Structure Adjustment," which aims to restructure its industrial landscape. In general, the MPE in Shanghai, southern Jiangsu, and northern Zhejiang is chiefly driven by industrial upgrading policy. ② Environmental regulation policy. After the "Taihu cyanobacteria incident," Jiangsu Province has ramped up efforts to clean up polluting enterprises in southern Jiangsu. Zhejiang Province has similarly implemented policies such as the "263 Special Environmental Remediation Action" and the "811" Environmental Pollution Control Action. Moreover, Shanghai has introduced its "Clean Air Action Plan" to curb air pollution. ③Pair assistance policies. Jiangsu, Zhejiang, and Anhui provinces have successively introduced provincial pair assistance policies, such as Jiangsu Province's "north–south link assistance" policy, Zhejiang Province's "Mountain and Sea Cooperation" plan, Anhui Province's "cooperative construction of a modern industrial park in northern Anhui". These policies are designed to promote coordinated regional development, but they have also resulted in the MPE from developed areas to less developed areas, such as northern Jiangsu and northern Anhui. Therefore, policymakers need to pay close attention to and optimize these policies to ensure a sustainable and balanced regional development.

## Influencing factors of space MPE

### Estimation results of the benchmark model

The regression analysis on the entire sample was conducted in Model (1). It was found that several variables had a significant impact on the migration of heavily polluting enterprises. Among them, variables with high statistical and economic significance included environmental regulation factors, industrial upgrading policies, the presence of strong incentive officials in the source area, average land price, urban development intensity, geographical distance, and terrain factors such as difference and the location advantage of coastal and interprovincial borders (Table 3). It is worth noting that "ecological civilization construction" was introduced during the 18th National Congress of the Communist Party of China in 2012. Following this, governments at all levels have substantially strengthened their environmental regulations, leading to a significant increase in the frequency and number of polluting enterprise destinations. In this light, a time heterogeneity analysis was conducted based on the benchmark model, with the year 2012 as the boundary. Results indicated that the influence of the aforementioned factors before 2012 (Model 2) and after 2012 (Model 3) differed.

1. Policy and official factors. Industrial upgrading policy exhibited consistently high significance level differences across regions. The difference in the intensity of environmental regulation indicates that the "pollution haven effect" is not universal, and only became statistically significant after 2012. Highly incentivizing officials in the source area are more likely to promote the MPE. Compared with the existing studies, which mainly observed the environmental regulatory factors driving MPE statically^12,14,15^, this study focuses on the dynamic changes of environmental regulatory factors. However, after 2012, highly incentive-oriented officials in the destination area will promote the settlement of polluting enterprises, which needs to be carefully monitored. Under the overall improvement of environmental regulations, less developed areas will have more opportunities to attract polluting enterprises, which may become a means for officials in those areas to be promoted. Additionally, paired assistance policies were found to be statistically significant at the 5% level in promoting the MPE. This is related to the corresponding counterparts in Jiangsu and Zhejiang provinces, who provide support to the MPE' spatial presence.
2. Industrial development environment. Prior to 2012, the urban development intensity was identified as the primary driver of such migration. However, a shift in the migration patterns of polluting enterprises occurred after 2012, which was predominantly influenced by profitability and cost factors. Specifically, the migration decisions were shaped by considerations of land cost, labor costs, and industrial agglomeration. These findings indicate that the MPE was previously impeded by constraints related to land resources, while post-2012, the migration decisions were predominantly driven by the profit and cost considerations of the enterprises.
3. Geographical location. Prior to 2012, inter-provincial boundary cities had a greater tendency to acquire larger areas, which corresponded with the findings of previous studies on the "boundary effect". However, after 2012, two distinct variables, namely geographic distance and Port advantage, emerged as significant predictors of the migration patterns of polluting enterprises. Port cities were found to possess substantial advantages in terms of import and export activities, as well as greater convenience for pollutant discharge, thus emerging as a vital alternative location for such enterprises.

**Table 3:** Baseline model and temporal heterogeneity analysis.

Type	Variable	(1)	(2)	(3)
		Full sample	Before 2012	After 2012
Policy and official factors	Environmental regulation	− 0.001***	− 0.000	− 0.001***
		(− 4.903)	(− 0.533)	(− 3.688)
	Industrial upgrading policy	− 3.021***	− 6.311**	− 5.244**
		(− 3.554)	(− 2.194)	(− 2.504)
	The characteristics of strong incentive officials from origin	0.107**	0.011	0.322***
		(2.001)	(0.115)	(4.186)
	The highly motivated official characteristics of the destination	0.003	− 0.085	0.181***
		(0.070)	(− 1.097)	(2.763)
	Pair assistance	0.778**	− 0.714	− 0.379
		(2.242)	(− 0.826)	(− 0.390)
Industry development environment	Total output value of industry above designated size	− 0.000**	− 0.000	0.000
		(− 2.069)	(− 0.276)	(0.658)
	Average unit price of land sale	− 0.970***	− 0.567	− 0.669***
		(− 8.213)	(− 1.230)	(− 4.521)
	Labor cost	− 0.117**	0.166	− 0.110*
		(− 2.458)	(1.319)	(− 1.682)
	Regional export intensity	1.562**	0.575	0.142
		(2.431)	(0.406)	(0.075)
	Industrial agglomeration	− 0.213*	− 0.284	− 0.683***
		(− 1.688)	(− 0.623)	(− 2.718)
	Urban development intensity	− 1.545***	− 3.540*	− 0.651
		(− 3.078)	(− 1.705)	(− 1.109)
	Regional innovation ability	− 0.139	− 0.252	− 0.212
		(− 1.036)	(− 0.548)	(− 0.687)
Geographical location	Geographical distance	− 0.005***	0.004	− 0.006***
		(− 4.740)	(0.363)	(− 3.089)
	Whether in this province	− 0.564*	− 12.006	− 0.062
		(− 1.819)	(− 0.023)	(− 0.113)
	Cultural similarity	− 0.053	0.361	0.011
		(− 0.185)	(0.261)	(0.019)
	Terrain difference	− 0.197***	− 0.228	− 0.169
		(− 3.213)	(− 0.596)	(− 1.386)
	Port advantage	0.612***	0.551	0.761*
		(3.079)	(0.854)	(1.717)
	Inter-provincial boundary city	0.682***	1.563*	0.707
		(3.063)	(1.876)	(1.503)
_cons		2.263***	13.406	2.460***
		(4.756)	(0.026)	(3.142)
N		10,864	4878	2950

*, ** and *** are significant at the statistical level of 10%, 5% and 1%, respectively, as in Table 4.

In conclusion, the location determinants of the spatial distribution of polluting enterprises have evolved from a primary focus on the scarcity of land resources to a more nuanced consideration of profitability factors such as land prices, labor costs, and industrial agglomeration. In addition, with respect to the geographical location aspect, there is a clear shift in preference towards coastal port cities as opposed to peripheral regions within a province.

### Industry heterogeneity analysis

In light of the intercorrelation between various industries, the manufacturing sectors of chemical raw materials, chemical products, rubber and plastic products, as well as pharmaceuticals, were integrated into a singular entity known as the "pharmaceutical and chemical industry". To further investigate this amalgamated industry, sub-sample estimation was carried out (Table 4).

**Table 4:** Analysis of industry heterogeneity.

Type	Variable	(4)	(5)	(6)	(7)
		Electric power heat Production and supply industry	Food manufacturing industry	Metal products industry	Pharmaceutical and chemical industry industries
Policy and official factors	Environmental regulation	− 0.001*	− 0.002***	− 0.000	0.000
		(− 1.888)	(− 3.196)	(− 0.897)	(0.472)
	Industrial upgrading policy	2.213	1.130	− 3.115	− 2.253
		(1.418)	(0.200)	(− 1.178)	(− 0.975)
	The characteristics of strong incentive officials from origin	0.308***	− 0.117	− 0.118	− 0.038
		(2.746)	(− 0.432)	(− 0.907)	(− 0.339)
	The highly motivated official characteristics of the destination	0.064	0.238	− 0.042	− 0.086
		(0.660)	(1.001)	(− 0.376)	(− 0.891)
	Pair assistance	1.066*	16.742	0.219	− 0.127
		(1.833)	(0.009)	(0.170)	(− 0.170)
Industry development environment	Total output value of industry above designated size	0.000**	0.000	− 0.000**	− 0.000***
		(1.969)	(0.005)	(− 2.445)	(− 3.005)
	Average unit price of land sale	− 0.616***	− 0.040	− 0.928***	− 0.919***
		(− 2.678)	(− 0.070)	(− 3.255)	(− 3.822)
	Labor costs	− 0.569***	− 0.478**	− 0.090	0.033
		(− 5.914)	(− 2.002)	(− 0.785)	(0.331)
	Regional export intensity	5.771***	− 3.852	1.064	2.680**
		(3.603)	(− 1.167)	(0.750)	(2.246)
	Industrial agglomeration	− 0.924***	1.250**	− 0.031	0.160
		(− 3.868)	(2.019)	(− 0.114)	(0.649)
	Urban development intensity	− 4.243***	− 2.278	0.300	− 0.134
		(− 3.388)	(− 0.686)	(0.287)	(− 0.155)
	Innovation ability	− 1.984***	− 0.507	0.301	0.283
		(− 6.972)	(− 0.714)	(0.973)	(1.071)
Geographical location	Geographical distance	− 0.003	− 0.004	− 0.004	− 0.012
		(− 1.592)	(− 0.347)	(− 1.088)	(− 1.487)
	Whether in this province	0.230	− 11.264	0.752	− 15.043
		(0.635)	(− 0.013)	(0.702)	(− 0.496)
	Cultural similarity	− 0.167	1.978	1.484	3.655
		(− 0.431)	(0.891)	(1.139)	(0.530)
	Terrain difference	− 0.054	0.101	− 0.097	0.054
		(− 0.715)	(0.090)	(− 0.386)	(0.056)
	Port advantage	0.865***	− 1.326	0.972	2.874
		(3.251)	(− 0.651)	(1.327)	(0.486)
	Inter-provincial boundary city	0.177	− 0.106	− 1.458	1.725
		(0.688)	(− 0.075)	(− 0.816)	(0.735)
_cons		− 1.808***	9.041	0.616	17.768
		(− 2.958)	(0.010)	(0.480)	(0.583)
N		5278	1414	4088	5012

Among various policy and official factors, it is found that environmental regulation policies have a greater impact on industries involved in power, thermal and food production industries. In addition, it has been observed that highly motivated officials in the country of origin can exert considerable pressure on enterprises operating in the power and thermal industries, leading them to withdraw from their local operations.

In the context of industrial development, regions with higher overall industrial output values are more appealing to the power and thermal generation industry, as such regions require the provision of power and thermal support for their local industrial enterprises. On the other hand, industries such as metal products, pharmaceuticals, and chemicals are more drawn to less developed regions with lower levels of industrial development. The impact of other industries' development environments varies across these four categories of industries. While the electric power and thermal generation enterprises are affected by various factors, the food manufacturing industry is primarily attracted by the advantage of lower labor costs. Meanwhile, the metal products, pharmaceutical, and chemical industries exhibit similar preferences, being attracted to areas with lower land costs. In terms of geographical location factors, it is mainly observed that the power and thermal industries are drawn to areas with port advantages.

## A typical case of the MPE: Nijiaxiang Group expanded from Wuxi to Yancheng

Jiangsu Nijiaxiang Group Co., LTD. (referred to as "Nijiaxiang Group"), located in Jiangyin City, Wuxi City, is a group enterprise involved in textile, printing and dyeing, fiber, chemical industry, trade logistics and other industries [Company Profile of Jiangsu Nijiaxiang Group Co., LTD. Can be obtained from its official website (http://www.neegroup.cn)]. It has established a holding subsidiary in Xiangshui County Jiangsu Tianjiayi Chemical Co., LTD. (referred to as Tianjiayi Company). In 2019, the company was hit by a catastrophic explosion known as the "March 21" incident, prompting the State Council to form an investigative team that disclosed a wealth of information about the group and the local government. This information facilitated an in-depth analysis of the factors that influenced Nijiaxiang Group's decision to invest in Tianjiayi Company, from Wuxi to Yancheng.

### Comparative analysis of the migration of origin and destination

From the perspective of Wuxi, the primary drivers for the outward MPE, including Nijiaxiang Group, are environmental regulations and the need to improve enterprise operating costs. Nijiaxiang Group, which is headquartered in Wuxi, traces its origins back to the "Jiangyin County Zhouzhuang Knitting Dyeing and Spinning Factory", a village-run collective enterprise established in 1979. From its inception, such enterprises have been characterized by insufficient technology, small scale, and significant pollution. Although they have grown alongside the southern Jiangsu economy, they have also been responsible for severe chemical pollution. In 2006, Jiangsu Province introduced the Special Rectification Plan of Chemical Production Enterprises, which aimed to complete the special rectification work of chemical production enterprises within three years. In 2007, the cyanobacteria crisis that erupted in Taihu Lake hastened the renovation of chemical enterprises. As a result, numerous polluting enterprises in Suzhou, Wuxi, and Chang areas were closed down and relocated. Nijiaxiang Group's environmental behavior information in Yancheng during its migration, prior to the open evaluation results, was marked as red.

Upon comparing the indicators of Wuxi and Yancheng, several notable differences emerge, including environmental regulation, economic aggregate, enterprise operation, and the carrying capacity of polluting enterprises (Table 5). Specifically, Yancheng has lower environmental regulation intensity, land and labor costs, and land development intensity compared to Wuxi. Additionally, Yancheng's flat terrain and coastal location offer favorable conditions for enterprises seeking to reduce their operating costs.

**Table 5:** Comparison of some indicators between Wuxi and Yancheng in 2006.

Variable type	Specific indicators	Unit	Origin (Wuxi)	Destination (Yancheng)	D-value
Policy factors	Comprehensive emission coefficient	\	18.51	22.39	3.88
	Industrial upgrading policy	\	82.23%	85.86%	3.64%
Industry development environment	Total output value of industry above designated size	RMB ¥ 100 million	7115.29	1378.14	− 5737.15
	Average unit price of land sale	Ten thousand yuan / ha	648.01	252.00	− 396.01
	Labor cost	Ten thousand yuan / year	2.96	1.56	− 1.40
	Export of goods	US $100 million	214.39	10.61	− 203.78
	Number of heavily polluting enterprises		10,490	6505	− 3985.00
	Urban development intensity	\	3.86%	0.41%	− 3.45%
	Number of heavily polluting enterprises		548	48	− 500
Industry development environment	Inter-provincial boundary city	\	1	1	0
	Coastal city	\	0	1	1
	Mean inclination	\	0.45	0.04	− 0.41
	Dialect area	\	The Wu dialect	Jianghuai mandarin	\

### Regionalism factors of the migration destination

The subsequent explanations outline the procedures to attract the polluting enterprises during the course of their selection and operation of business sites.

Firstly, the pressure of economic migration and the absence of ecological preservation consciousness. In the quest to augment industrial productivity, certain regional bureaucrats advocate the viewpoint that "they would rather die from contamination than from destitution." Consequently, a profusion of contaminating enterprises previously ousted from Zhejiang and southern Jiangsu are assimilated. The director of the Environmental Protection Bureau in Xiangshui County, designated as K, posits that, "When faced with the choice between sustenance and habitation or environmental preservation, people would undoubtedly choose the former. This is not indicative of our inaptitude, but rather due to the absence of alternative choices."

Secondly, regional officials transgress discipline and regulations by furnishing amenities to polluting enterprises. During the phase of business location selection, in 2006, governmental officials in Liutao Township of Xiangshui County acted as representatives for Tianjiayi Company in securing project approvals, business licenses, and related procedures. Additionally, in the course of enterprise operations, local government officials failed to oversee and indulged illegal construction by the enterprises. Prior to approval, Tianjiayi Company undertook six batches of projects. Moreover, certain officials in Xiangshui County "colluded with officials and businessmen from multiple" small chemical "enterprises and traded power and wealth", and facilitating the illegal conduct of the enterprises.

Thirdly, the locality possesses an expansive "environmental capacity" and favorable drainage conduits. Xiangshui County's Ecological Chemical Industry Park, the whereabouts of Tianjiayi Company, is favorably situated for sewage discharge, given its proximity to the Guanhe River in the north and the Yellow Sea in the east. The Guanhe River functions as a watercourse for several rivers in northern Jiangsu to enter the sea, and it constitutes the most extensive tidal river in northern Jiangsu to enter the sea. As indicated in the materials for attracting local investments, "Guanhe River features a substantial tidal drop, a mean river breadth of 1500 m, formidable self-purification capability, and a sizable environmental capacity."

These micro factors have diminished the extent of regional environmental governance from diverse perspectives, and propelled Xiangshui to metamorphose into the "haven" of Nijiaxiang Group and other contaminating enterprises, thereby supplying tangible substantiation for the "pollution haven" hypothesis.

## Conclusion and discussion

### Research conclusion

This study delved into the MPE and its influencing factors by analyzing investment data of enterprises from Qichacha. The main findings obtained from the study are summarized as follows:

1. According to the migration path perspective, the polluting enterprises situated in the Yangtze River Delta underwent an evolutionary process: "adjacent migration—intra-provincial adjacent migration to intra-provincial long-distance migration—emphasizing intra-provincial migration, supplemented by inter-provincial migration—parallel intra-provincial and inter-provincial migration". Concerning the city level, the primary source of polluting enterprise migration was Shanghai, followed by Nanjing, Suzhou, Hangzhou, Hefei, and other provincial capitals or central cities. Meanwhile, the less developed regions, such as northern Jiangsu and northern Anhui, served as the "pollution haven".
2. According to the estimated results of the panel negative binomial regression model, the geographical factors that contribute to the MPE are diverse. These factors range from industrial upgrading to profitability considerations, such as land price, labor cost, and industrial agglomeration. Additionally, the migration trend has shifted from inter-provincial marginal regions to coastal port cities.
3. Through a case analysis of the migration of the "Nijiaxiang Group", it is evident that there exist several micro-level factors that contribute to the MPE. These factors include deficiencies in environmental protection concepts, official corruption, and inadequate rule of law and social governance. As a result, the local environmental regulation level has been compromised from various perspectives, creating a "haven" for equal-polluting enterprises. This case provides valuable empirical evidence in support of the "pollution haven" hypothesis.

The primary objective of this research paper is to investigate the utilization of inter-enterprise investment relationship data, conduct a comprehensive analysis of the internal structure of "group enterprises," and scrutinize the migration behavior of polluting enterprises from the perspective of "inter-regional association." Previous studies have primarily concentrated on the evolution of industrial geographical patterns, but have failed to reveal the internal enterprise liquidity^2^. Alternatively, these studies have categorized polluting enterprises into entry and exit behaviors and have focused on "local" factors^4^. However, this research paper has utilized unique research methods and data to address the aforementioned research gaps. Furthermore, this study provides evidence that the "pollution haven hypothesis" resulting from environmental regulations is not globally applicable, but only observed in the Yangtze River Delta after 2012.

### Policy advice and discussion

The empirical findings of this research paper have significant policy implications: (1) We found that there are differences in environmental regulation policies in the Yangtze River Delta region, which can affect MPE. Therefore, policymakers should establish an environmental regulation system that focuses on fairness and justice, thereby preventing polluting enterprises from relocating due to differences in environmental regulations. (2) The environmental monitoring mechanism of pair assistance policies should be improved, as polluters are expanding into less developed areas under these policies. Jiangsu Province and Zhejiang Province in the Yangtze River Delta region are among the earliest in China to carry out the exploration of pair assistance policies. Pair assistance policies, which were originally designed to enhance economic and social development in underdeveloped regions, have now become transmission channels for pollutants and polluting enterprises, necessitating special attention. Therefore, it is essential to strengthen the environmental monitoring mechanism when formulating and implementing pair assistance policies. (3) The source of polluting enterprises should be considered as one of the bases for ecological compensation and technological compensation. A lifelong environmental tracking and monitoring system is expected to be established for polluting enterprises and their stakeholders, based on the Superfund Act of the United States and the "Dark Green" revolution of the four Nordic countries.

This paper is not without limitations that necessitate further elaboration in future research efforts. Firstly, the analysis of both the comprehensive MPE and the internal migration of cities divided into districts requires further attention. Secondly, the extant research pertaining to the identification of polluting enterprises primarily rests on the scrutiny of 2-digit industry categories, thus lacking in granularity. Given the disparities in technological advancement within industries, particularly in the context of the value chain division of labor, there may be significant variation in pollution emission levels despite belonging to the same industry classification. Accordingly, forthcoming research will leverage enterprise-level pollution emission data to explore the degree of pollution emission intensity among different value chain links.

## Data Availability

The datasets generated and analyzed during the current study are not publicly available due the author is also working with other scholars to carry out other related studies but are available from the author on reasonable request. For scholars who require data, please contact lbq0802@163.com.
